# Tissue Damage Markers after a Spinal Manipulation in Healthy Subjects: A Preliminary Report of a Randomized Controlled Trial

**DOI:** 10.1155/2014/815379

**Published:** 2014-12-25

**Authors:** A. Achalandabaso, G. Plaza-Manzano, R. Lomas-Vega, A. Martínez-Amat, M. V. Camacho, M. Gassó, F. Hita-Contreras, F. Molina

**Affiliations:** ^1^Centro de Fisioterapia y Psicología Soluciona, 18002 Granada, Spain; ^2^Department of Medicine, Universidad Complutense de Madrid, 28040 Madrid, Spain; ^3^Department of Health Sciences, Universidad de Jaén, Paraje Las Lagunillas s/n, 23071 Jaén, Spain; ^4^Servicio de Análisis Clínicos, Hospital Médico-Quirúrgico del Complejo Hospitalario de Jaén, Servicio Andaluz de Salud, 23007 Jaén, Spain

## Abstract

Spinal manipulation (SM) is a manual therapy technique frequently applied to treat musculoskeletal disorders because of its analgesic effects. It is defined by a manual procedure involving a directed impulse to move a joint past its physiologic range of movement (ROM). In this sense, to exceed the physiologic ROM of a joint could trigger tissue damage, which might represent an adverse effect associated with spinal manipulation. The present work tries to explore the presence of tissue damage associated with SM through the damage markers analysis. Thirty healthy subjects recruited at the University of Jaén were submitted to a placebo SM (control group; *n* = 10), a single lower cervical manipulation (cervical group; *n* = 10), and a thoracic manipulation (*n* = 10). Before the intervention, blood samples were extracted and centrifuged to obtain plasma and serum. The procedure was repeated right after the intervention and two hours after the intervention. Tissue damage markers creatine phosphokinase (CPK), lactate dehydrogenase (LDH), C-reactive protein (CRP), troponin-I, myoglobin, neuron-specific enolase (NSE), and aldolase were determined in samples. Statistical analysis was performed through a 3 × 3 mixed-model ANOVA. Neither cervical manipulation nor thoracic manipulation did produce significant changes in the CPK, LDH, CRP, troponin-I, myoglobin, NSE, or aldolase blood levels. Our data suggest that the mechanical strain produced by SM seems to be innocuous to the joints and surrounding tissues in healthy subjects.

## 1. Introduction

Spinal manipulation (SM) is a common form of intervention used by a wide range of practitioners used to relieve pain and disability of the musculoskeletal system. A precise definition of SM is still under review. The SM is frequently defined as a manual procedure that involves a directed impulse to move a joint past its physiologic ROM without exceeding its anatomical limit [1, 2]. Although its effectiveness has been demonstrated in some spinal syndromes such as [3, 4], several studies show concomitant organic complications with the application of cervical spinal manipulation (CSM) [2, 5, 6]. Mild to moderate adverse effects occur in a large proportion of patients receiving spinal manipulation [7]. Although the majority of the adverse effects are transient and nonserious [7], severe adverse events such as cerebrovascular accidents and paraplegia have been associated with SM [8]. These injuries are typically described following an upper-back CSM. Although some authors have proposed that, in the presence of a marked degree of atherosclerosis, the mechanical stretching and compression effects of rotational manipulative techniques [9] may impose a further risk factor not only for vertebrobasilar insufficiency but also for lesions of the endothelium [10], recent studies suggest that SM may induce less arterial strain than the range of motion test when cervical rotation is examined [11].

Therefore, there are controversial data about the possibility of CSM inducing pathological mechanical stress that might in turn provoke vascular and neurological accidents [11–14]. Some evidence supports thoracic spinal manipulation (TSM) as an alternative to CSM to relieve pain and disability in the cervical spine [15]. There is no evidence of serious adverse events related to TSM. Given this situation, it is necessary to determine whether spinal manipulation is an innocuous technique.

Several proteins have been widely used in medicine as markers of tissue damage. These damage biomarkers are cell proteins or enzymes normally located inside specific cells. The detection of these proteins in serum and cerebrospinal fluid is a tell-tale of cell breakage produced by tissue damage. Proteins like creatine phosphokinase (CPK), lactate dehydrogenase (LDH), aldolase, myoglobin, and troponin-I [16] have been described as tissue damage markers in conditions such as strenuous exercise, brain injury, and heart damage [16–21], when altered levels of theses markers were detected in blood samples. CPK [22] and myoglobin [16] are the most widely used blood parameters for the detection of striated muscle injury although other parameters seem to be more sensitive to the difference between cardiac muscle and skeletal muscle damage. In this respect, the skeletal muscle troponin-I subunit may be an earlier and more suitable marker for skeletal muscle damage than CPK [23] and it is widely used as a muscle damage maker after strenuous exercise [24].

On the other hand, neuron-specific enolase (NSE) is also a glycolytic enzyme, which occurs in neurons and axons and is an appropriate marker for neuronal damage [25].

Finally, C-reactive protein (CRP) is a widely employed systemic marker of inflammation and tissue damage [26].

The aim of this preliminary study is to determine the possible noxious effects of spinal manipulation. To the extent of our knowledge, this is the first work that focused on the study of SM and mechanically induced tissue damage through the analysis of damage biomarkers in blood samples.

## 2. Methods

### 2.1. Design Overview

It is randomized repeated-measures controlled trial.

### 2.2. Setting and Participants

The study was approved by the Ethics Committee of the University of Jaén (Spain), and all subjects provided written informed consent. A total of 40 healthy students from the University of Jaén were previously selected for this study. Subjects who had one or more of the following conditions were excluded from the current study: contraindication to manipulation, history of whiplash or cervical surgery, pain related to cervical spine or arm in the previous month, subject under pharmacological treatment, subject who has practice strenuous exercise 7 days prior to the experiment, headache in the previous days, having undergone spinal manipulative therapy in the previous 2 months, or loss of standing balance. Information about eating and physical exercise habits was obtained from participants after the experiment was conducted.

### 2.3. Randomization and Intervention

A number was assigned to each participant by an external consultant, who did not have any further involvement in the research. After that, a list of random numbers ranging from 0 to 30 was generated by Microsoft Excel software. The first ten numbers of this list were assigned to the control group, the next ten numbers to the cervical manipulation group, and the last ten to the thoracic manipulation group. Randomization was done by an external assessor who did not participate in the research.

Thoracic SM technique involved a high-velocity, end-range, anterior-posterior force through the elbows to the middle thoracic spine (T3-T4) on the lower thoracic (T4-T5) spine in a supine position with patient's arms crossed. In the cervical manipulation, a high-velocity, midrange left rotational force to the midcervical spine (C4) on the lower (C5) cervical spine in supine, with left rotation and right side bending.

Control participants were treated following the cervical manipulation protocol with regard to hand contact, but without intention of mobilization, nor application of tissue tension by the treating clinician.

The time devoted to HVLA thrust manipulations in the intervention groups and that to the simulated procedures in control group were similar, in order to minimize the potential for an attention effect. All the researchers were blinded to the therapist's intervention.

### 2.4. Blood Extraction

Serum samples were extracted before intervention, right after intervention, and two hours after manipulation by venipuncture of the cephalic vein using a Vacutainer system (Becton-Dickinson, United Kingdom). Blood was collected in two different tubes for both serum (BD Vacutainer SST II Advance, ref. 367953) and plasma (BD Vacutainer LH PST II Advance, ref. 367374) separation. After blood extraction, tubes were let stand at room temperature for one hour until the blood clotted. Afterwards, tubes were centrifuged for ten minutes at 2000 g (Avanti J-30I, Beckman Coulter, USA). Supernatant was collected from the tubes. Three serum aliquots were done for the determination of CPK, LDH, CRP, aldolase, and NSE. Plasma was divided into two different aliquots for troponin-I and myoglobin assay. All the aliquots were kept at −80°C until used.

### 2.5. Outcomes and Follow-Up

CPK, LDH, and aldolase serum concentrations were calculated by enzymatic assay in an OLYMPUS AU5400 Analyzer (Beckman-Coulter, USA). Troponin-I levels were measured through chemiluminescence (Dimension EXL, Siemens, Germany), as was neuron-specific enolase (LiasonAnalyzer, Dia-Sorin). CRP concentration was determined by turbidimetry assay (OLYMPUS AU5400 Analyzer, Beckman Coulter, USA). Myoglobin was determined by means of enzymatic immunoassay (Dimension EXL, Siemens, Germany). All the assays were carried out in the Ciudad de Jaén University Hospital following the manufacturer's protocol.

### 2.6. Statistical Analysis

Continuous variables were described by means and standard deviation, and categorical variables by frequencies and percentages. Kolmogorov-Smirnov was used to verify the normal distribution of continuous variables in the groups, and Levene's test was used to confirm the homoscedasticity of the samples. One-way analysis of variance and Chi-squared test were used to prove comparability on sociodemographic baseline values. To prove the effect of the independent factor (control, thoracic, or cervical manipulation) on the dependent variables (blood concentrations) at each time point (pretreatment, zero hours posttreatment, and two hours posttreatment), a mixed-model ANOVA was employed. The hypothesis of interest was the group-by-time interaction. A separate 3 × 3 mixed-model ANOVA was applied for each dependent variable. In order to measure the effect size, eta-squared and Cohen's *d* were used for group-by-time effect and pairwise comparisons, respectively. For Cohen's *d* interpretation an effect size of 0.2 was considered small, 0.5 moderate, and 0.8 large [27]. Demographic and experimental data were treated with the software SPSS 19.0 (IBM, USA) and MedCalc12.7 (MedCalc, Belgium). All of the analyses were performed with a 95% confidence interval (*P* < 0.05).

## 3. Results

**Figure 1:**
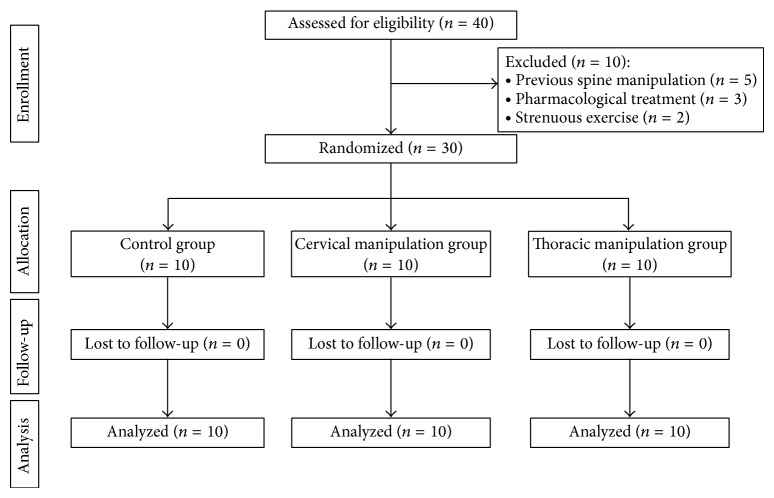
Flowchart diagram of the study.

Of the 40 patients screened in the University of Jaén, a total of 30 subjects met the inclusion criteria and agreed to participate in the study. Ten participants were randomly assigned to each treatment group. Demographic data are displayed in Table 1.  Figure 1 shows the flowchart depicting participant recruitment and retention. No differences were observed between groups at baseline measures. Only CPR shows a difference in the limits of significance. No vitamin supplementation was reported by the participants. They followed the Mediterranean diet [28] and had a sedentary lifestyle [29].

Descriptive data for all dependent variables in each group for each time point are shown in Table 2. Mixed-model ANOVA failed to reveal a group-by-time interaction in any of the dependent variables (*P* > 0.05). Effect sizes, measured with eta-squared, were small for all dependent variables and the interaction never explained more than 12% of the variance (Table 2). The higher effect was apparent for LDH (11.2%) and the smaller was shown for aldolase (1.9%).

The pairwise comparisons between control and both the thoracic and cervical manipulation groups show a result in the limits of statistical significance only for the comparison between control and thoracic groups in CRP at zero hours posttreatment and for myoglobin at two hours posttreatment (Table 3). However, these statistical significances have no real meaning because of the lack of statistical significance of the ANOVA. Nevertheless, the effect sizes could be considered high (*d* > 0.8) for the comparison between the control and the thoracic groups for CRP in all time points and for myoglobin at two hours posttreatment. In the comparison between control and cervical manipulation groups, only the effect size for myoglobin at two hours posttreatment could be considered high (Table 3). Troponin-I data are not shown because the value for the subjects was zero in all the time-point and groups.

## 4. Discussion

Spine manipulation (SM) is a manual therapy technique commonly applied, which presents benefits for patients such as an anti-inflammatory effect [30], pain relief, and reduction of drug consumption [31]. However, some studies have proposed a noxious effect of SM application. In this sense, the reports on its effects on inducing tissue injuries are controversial, as it has been related to adverse events [7, 12, 32, 33]. The literature points at catastrophic manual-therapy-induced adverse events being dependent on tissue damage. In this sense, the detection of some proteins in blood samples has been revealed as useful in some musculoskeletal and neurological conditions [34–36] to detect tissue damage. Thus, the present work is focused on the determination of biological damage markers in blood samples after a cervical or a thoracic manipulation, in order to corroborate whether or not spinal manipulation causes measurable tissue damage.

Adverse events from manual therapy range from the catastrophic, such as cervical artery dissection producing a stroke, through bruising to muscle soreness that could be regarded as a minor, and expected, consequence of treatment [37]. Rubinstein et al. reported that 72% of adverse events occurred after the first treatment [38]. Most adverse events reported by manual therapy patients are thought to be benign and transient and are often unknown to the practitioner unless patients show observable signs (e.g., loss of motion or neurological deficits) or report pain or discomfort [39]. A recent systematic review shows that nearly half of patients experience adverse events after manual therapy. These adverse events are short-lived and minor, and most will occur within 24 hours and resolve within 72 hours. The relative risk of these adverse events is similar for manual therapy plus exercise treatment and for sham/passive/control interventions [37].

The biomarkers used in the study are generally used to detect tissue damage. Creatine phosphokinase (CPK) is an intracellular enzyme related to energy metabolism, and its level in serum has been extensively used as a diagnostic marker for muscle injury [18]. Myoglobin and troponin-I are sensitive markers for skeletal muscle or cardiac muscle damage [19]. Both aldolase and lactate dehydrogenase (LDH) are biomarkers for general tissue damage. The presence of neuron-specific enolase (NSE) in plasma samples is used as a diffuse neuronal damage marker [40]. The C-reactive protein (CRP) is a nonspecific marker of inflammation. In fact, Huang et al. showed that elevated mechanical strain on vessels could induce the local expression of proinflammatory cytokines like CRP [41]. These parameters had been extensively used in research in order to analyze the effect of active physical therapy in the expression of tissue damage markers [42, 43]. After the analysis of seven tissue damage markers, our data do not show any significant differences in CPK, LDH, troponin-I, myoglobin, aldolase, NSE, and CRP concentrations. A mixed-model ANOVA failed to reveal a group-by-time interaction in any of the dependent variables (*P* > 0.05). The pairwise comparisons between the control and both the thoracic and the cervical manipulation groups show lack of statistical significance except for two comparisons in the limit of statistical significance (which lack real meaning). The relatively high effect size found in the comparison of CRP levels between the control group and the thoracic manipulation group was already apparent at pretreatment and did not increase significantly in the two posttreatment measurements. The behavior of these parameters suggests that the mechanical stimulus induced by spinal manipulation alone is not enough to provoke cell damage or tissue breakage in healthy subjects. These data agree with other works that show no alteration in pathologic blood vessels after a cervical manipulation [44]. Nevertheless, Huang et al. showed that mechanical strain increased CRP expression in the saphenous vein in a strength-dependent manner [41]. Rather than an episode of mechanical stretch, some studies propose that a repetitive mechanical stress was needed to induce microstructural damage in vessels [45]. Moreover, studies demonstrated that the mechanical load of the vertebral artery during SM application was almost an order of magnitude lower than the strain required to cause its mechanical disruption [14].

The findings of the present study contradict those of previous studies on other manual therapies modalities. Danneskiold-Samsøe et al. identified an increase on serum myoglobin after a deep massage [46, 47]. Arkko et al. also found increases of serum CPK and LDH concentrations [48]. This contradiction may be due to differences between modalities or because the studies of Danneskiold-Samsøe and Arkko were conducted on nonhealthy subjects whereas our subjects were healthy. Moreover, the deep massage protocol used in these studies differs from our manipulation technique in the application time of the treatment protocol. The process for a single spinal manipulation takes a few seconds but, in contrast, massage protocol takes some minutes. It is therefore possible that the long time during which mechanical force was directly applied to tissues could explain the observed increase in myoglobin, CPK, and LDH in these studies [4, 38, 39].

After the manipulation, two of the subjects suffered syncope. Both belonged to the thoracic manipulative group. It is likely that syncope after spinal manipulative therapy is not related to tissue damage and that such adverse event may be explained by other reasons.

*Strengths and Limitations of the Study*. First, the sample was small due to methodological issues, making it difficult to generalize our results. Second, it was not possible to blind the clinician or the subjects due to the nature of the intervention, which constitutes a risk of bias. Third, the present study was conducted on asymptomatic subjects, so it is not possible to extrapolate the present findings to a symptomatic population.

## 5. Conclusions

Our data show no changes in any of the studied damage markers. Although this study examined the outcomes in an asymptomatic population, lower cervical and thoracic manipulative techniques seem to be safe manual therapies techniques which cause no harm to the health of the subject. These data may be used as evidence of the safe application of spinal manipulation to healthy subjects. Further studies with a large sample size and a patient population are needed to corroborate the innocuous effects of spinal manipulation.

## Figures and Tables

**Table 1:** Sociodemographic characteristics and baseline measures of the groups.

Characteristic	Control *n* = 10	Thoracic *n* = 10	Cervical *n* = 10					*P*-value
Age^*^ (years)	27.60 ± 3.22	29.80 ± 3.52	28.60 ± 3.99					0.195
BMI^*^	21.45 ± 2.38	23.98 ± 3.97	23.17 ± 2.94					0.206
Weight^*^ (Kg)	66.60 ± 8.47	73.70 ± 14.33	71.20 ± 12.19					0.528
Height^*^ (m)	1.72 ± 0.07	1.75 ± 0.06	1.75 ± 0.12					0.301
CPK^*^ (U/L)	74.90 ± 17.21	72.00 ± 13.73	65.10 ± 14.78					0.353
LDH^*^ (U/L)	276.03 ± 57.30	297.20 ± 52.66	275.90 ± 43.40					0.573
Enolase^*^ (ng/mL)	9.48 ± 1.81	9.09 ± 1.50	9.57 ± 3.26					0.887
CPR^*^ (mg/L)	1.41 ± 1.00	0.56 ± 0.30	1.48 ± 1.16					0.056
Aldolase^*^ (U/L)	3.09 ± 1.60	3.10 ± 1.06	3.13 ± 1.02					0.998
Myoglobin^*^ (ng/mL)	50.76 ± 31.39	36.80 ± 10.81	35.40 ± 20.87					0.262
Gender^†^	Male	6	60.0%	5	50.0%	5	50.0%	0.875
	Female	4	40.0%	5	50.0%	5	50.0%	

BMI: body mass index; CPK: creatine phosphokinase; LDH: lactate dehydrogenase; CRP: C-reactive protein.

^*^Continuous variables are expressed as a mean ± standard deviation. *P* values correspond to one-way ANOVA test.

^†^Categorical variables are expressed as frequencies and percentages. *P* values correspond to Chi-squared test.

**Table 2:** Blood concentrations for all groups at each follow-up period. Statistical significance and effect size for group-by-time interaction.

		Pre-T	Post-0 H	Post 2 H	*P* value	Eta^2^
		Mean ± SD	Mean ± SD	Mean ± SD		
CPK (U/L)	Control	74.90 ± 17.21	74.10 ± 17.12	74.40 ± 16.57	0.425	0.065
	Thoracic	72.00 ± 13.73	72.10 ± 16.00	71.10 ± 19.09		
	Cervical	65.10 ± 14.78	65.20 ± 16.29	68.10 ± 16.38		

LDH (U/L)	Control	276.03 ± 57.30	283.43 ± 44.29	268.11 ± 47.02	0.167	0.112
	Thoracic	297.20 ± 52.66	294.70 ± 52.69	289.80 ± 67.36		
	Cervical	275.90 ± 43.40	276.00 ± 28.20	302.80 ± 82.64		

Enolase (ng/mL)	Control	9.48 ± 1.81	8.90 ± 1.24	8.86 ± 1.29	0.235	0.100
	Thoracic	9.09 ± 1.50	9.52 ± 1.41	9.42 ± 2.95		
	Cervical	9.57 ± 3.26	9.23 ± 1.97	11.57 ± 6.17		

CPR (mg/L)	Control	1.41 ± 1.00	1.61 ± 1.02	1.53 ± 1.19	0.486	0.058
	Thoracic	0.56 ± 0.30	0.50 ± 0.27	0.50 ± 0.30		
	Cervical	1.48 ± 1.16	1.48 ± 1.22	1.47 ± 1.15		

Aldolase (U/L)	Control	3.09 ± 1.60	3.36 ± 1.37	3.50 ± 1.57	0.859	0.019
	Thoracic	3.10 ± 1.06	3.23 ± 1.25	3.17 ± 1.37		
	Cervical	3.13 ± 1.02	3.29 ± 0.90	3.63 ± 1.38		

Myoglobin (ng/mL)	Control	50.76 ± 31.39	52.18 ± 23.63	70.20 ± 43.56	0.312	0.083
	Thoracic	36.80 ± 10.81	38.30 ± 10.80	36.60 ± 10.60		
	Cervical	35.40 ± 20.87	35.80 ± 19.62	39.10 ± 22.19		

Pre-T: pretreatment values; Post-0 H: values 0 H after intervention; Post-2 H: values 2 H after intervention; CPK: creatine phosphokinase; LDH: lactate dehydrogenase; CRP: C-reactive protein.

**Table 3:** Mean differences between control and both the thoracic and the cervical groups.

		Control-thoracic				Control-cervical			
		Mean difference	95% CI	*P* value	Cohen-*D*	Mean difference	95% CI	*P* value	Cohen-*D*
CPK (U/L)	Pretreatment	2.90	(−14.57; 20.37)	1.000	0.19	9.80	(−7.67; 27.27)	0.491	0.61
	Post 0 H	2.00	(−16.81; 20.81)	1.000	0.12	8.90	(−9.91; 27.71)	0.713	0.53
	Post 2 H	3.30	(−16.55; 23.15)	1.000	0.18	6.30	(−13.55; 26.15)	1.000	0.38

LDH (U/L)	Pretreatment	−21.17	(−79.90; 37.55)	1.000	0.38	0.13	(−58.60; 58.85)	1.000	0.00
	Post 0 H	−11.27	(−60.29; 37.75)	1.000	0.23	7.43	(−41.59; 56.45)	1.000	0.20
	Post 2 H	−21.69	(−98.48; 55.11)	1.000	0.37	−34.69	(−111.48; 42.11)	0.777	0.52

Enolase (ng/mL)	Pretreatment	0.39	(−2.26; 3.04)	1.000	0.23	−0.09	(−2.74; 2.56)	1.000	0.04
	Post 0 H	−0.62	(−2.42; 1.17)	1.000	0.47	−0.33	(−2.13; 1.46)	1.000	0.20
	Post 2 H	−0.56	(−5.15; 4.03)	1.000	0.24	−2.71	(−7.30; 1.88)	0.431	0.61

CRP (mg/L)	Pretreatment	0.85	(−0.18; 1.88)	0.136	1.14	−0.07	(−1.10; 0.96)	1.000	0.07
	Post 0 H	1.11	(0.05; 2.18)	**0.039**	1.49	0.13	(−0.93; 1.20)	1.000	0.12
	Post 2 H	1.03	(−0.09; 2.14)	0.078	1.18	0.06	(−1.06; 1.17)	1.000	0.05

Aldolase (U/L)	Pretreatment	−0.01	(−1.44; 1.43)	1.000	0.00	−0.04	(−1.47; 1.40)	1.000	0.03
	Post 0 H	0.13	(−1.23; 1.49)	1.000	0.10	0.07	(−1.29; 1.43)	1.000	0.06
	Post 2 H	0.33	(−1.32; 1.98)	1.000	0.22	−0.13	(−1.78; 1.52)	1.000	0.09

Myoglobin (ng/mL)	Pretreatment	13.96	(−11.89; 39.80)	0.538	0.59	15.36	(−10.49; 41.20)	0.423	0.58
	Post 0 H	13.88	(−7.57; 35.34)	0.331	0.76	16.38	(−5.07; 37.84)	0.185	0.75
	Post 2 H	33.60	(0.63; 66.57)	0.045^*^	1.06	31.10	(−1.87; 64.07)	0.069	0.90

Pre-T: pretreatment values; Post-0 H: values 0 H after intervention; Post-2 H: values 2 H after intervention; CPK: creatine phosphokinase; LDH: lactate dehydrogenase; CRP: C-reactive protein.

^*^Trend to statistical significance.
